# Ginsenoside Rh2 attenuates the progression of non‐small cell lung cancer by sponging miR‐28‐5p/STK4 axis and inactivating Wnt/β‐catenin signaling

**DOI:** 10.1002/cam4.5960

**Published:** 2023-04-20

**Authors:** Jun Ma, Di Zhao, Dahai Yu, Wei Song, Xiaofang Yang, Haitao Yin

**Affiliations:** ^1^ Radiotherapy Department Affiliated Hospital of Nanjing University of Chinese Medicine China; ^2^ Radiotherapy Department Xuzhou Central Hospital China

**Keywords:** ginsenoside Rh2 (G‐Rh2), miR‐28‐5p/STK4 axis, non‐small cell lung cancer (NSCLC), tumor growth, Wnt/β‐catenin signaling

## Abstract

**Background:**

Ginsenoside Rh2 (G‐Rh2) exerts anti‐tumor activity in non‐small cell lung cancer (NSCLC). microRNAs (miRNAs, miRs) play pivotal roles in NSCLC. We aimed to investigate whether G‐Rh2 inhibited NSCLC progression by targeting miRNA.

**Methods:**

Cell viability, apoptosis and cycle were determined by Cell Counting Kit‐8, 6‐diamidino‐2‐phenylindole (DAPI) staining and flow cytometry. The potential target miRNAs of G‐Rh2 were screened by real‐time quantitative polymerase chain reaction (RT‐qPCR). The difference in miR‐28‐5p expression between lung adenocarcinoma (LUAD) tissues and normal tissues or lung squamous cell carcinoma (LUSC) tissues and normal tissues was retrieved from TCGA‐LUAD and TCGA‐LUSC, respectively. Kaplan–Meier Plotter was conducted to analyze the survival rate for different serine/threonine‐protein kinase 4 (STK4) expressions with different prognostic risks. immunohistochemistry of STK4 expression in non‐tumor and tumor tissues was analyzed from the HPA database. RT‐qPCR and Western blot were adopted for detecting mRNA and protein expression. TargetScan V7.2, miRanda and PITA were adopted for predicting targets of miR‐28‐5p, overlapped genes were subjected to GO analysis. The interactions of miR‐28‐5p‐Wnt and miR‐28‐5p‐STK4 were detected by TOP/FOP luciferase reporter assay and dual luciferase reporter assay, respectively.

**Results:**

Current study observed that G‐Rh2 reduced miR‐28‐5p expression in NSCLC cells dose‐dependently. miR‐28‐5p was upregulated in NSCLC tissues and cells. The target genes of miR‐28‐5p were enriched in negative regulation of Wnt signaling. miR‐28‐5p inhibitor inactivated Wnt signaling, inhibited cell viability and cell cycle, while enhanced cell apoptosis of NSCLC cells by targeting STK4. G‐Rh2 exerted the similar effects with miR‐28‐5p inhibitor by reducing miR‐28‐5p. G‐Rh2 and miR‐28‐5p inhibitor exerted a synergistic effect on inhibiting NSCLC tumor growth.

**Conclusion:**

In conclusion, G‐Rh2 attenuates NSCLC development by affecting miR‐28‐5p/STK4 axis and inactivating Wnt signaling. Taken together, we project out a novel therapeutic target for NSCLC.

## 1 INTRODUCTION

In a study reported in 2021, lung cancer was reported to remain the most prevalent cause of cancer‐associated mortality in males and females; moreover, it was estimated that lung cancer led to approximately 1,800,000 deaths globally.^1^ Generally, lung cancer can be distinguished into two subtypes: non‐small cell lung cancer (NSCLC) and small cell lung cancer (SCLC).^2^ In contrast to SCLC, NSCLC is relatively more common, comprising more than 80% of lung cancer cases.^3^ The 5‐year overall survival (OS) rate of patients with NSCLC <10%,^4^ which attributes to its proliferative and aggressive phenotypes.^5^ In spite of the advances achieved in the treatment of NSCLC, deep investigations on exploring innovative therapeutic approaches are required to promote the prognosis of NSCLC.^6^

microRNAs (miRs) are a form of highly conserved, single‐stranded small non‐coding molecules (22 nt) in mammalian cells.^7^ miRNAs degrade mRNA or inhibit translation by binding with the 3′‐untranslated region (3′UTR) of target mRNAs.^8^ In addition, miRNAs participate in the regulation of multiple biological processes, for example, cell proliferation, apoptosis, migration, and invasion,^9^ moreover, their dysregulation contributes to the development of various cancer types, including NSCLC.^10^ Increasing miRNAs are affirmed to be key cancer‐related miRNAs in NSCLC, which function by targeting oncogenes or tumor suppressors.^11^, ^12^

As one of the bioactive components in ginseng, Ginsenoside Rh2 (G‐Rh2) serves as a traditional herbal medicine which is extensively utilized.^13^, ^14^ G‐Rh2 is well known to exert anti‐tumor activity against numerous cancers,^15^ including prostate cancer,^16^ and hepatocellular carcinoma.^17^ Moreover, G‐Rh2 exert anti‐tumor activity against NSCLC, for example, G‐Rh2‐mediated G1 growth arrest and apoptosis in NSCLC cells A549,^18^ G‐Rh2 attenuates tumor migration and invasion by regulating the crosstalk between NSCLC and tumor‐associated macrophages.^19^ However, the precise molecular mechanisms which are responsible for the anti‐tumor effects of G‐Rh2 in NSCLC remain complicated. Recently, the interaction between G‐Rh2 and miRNAs has attracted more and more attentions. For instance, G‐Rh2 represses cell growth by regulation of miR‐4295/CDKN1A axis in prostate cancer,^16^ G‐Rh2 reduces cell proliferation and migration by downregulating miR‐31 in medulloblastoma.^20^ Moreover, numerous miRNAs were found to be regulated by G‐Rh2 by In‐Sook An et al, that is, 44 miRNAs were upregulated and 24 miRNAs were downregulated in a microarray assay in NSCLC cell line A549.^21^ However, the key miRNAs responsible for the function of G‐Rh2 have not been identified in NSCLC. Herein, we aimed to identify miRNAs which could be regulated by G‐Rh2 to attenuate the progression of NSCLC in vitro and in vivo, thus providing novel therapeutic approaches for NSCLC.

## 2 MATERIALS AND METHODS

### 2.1 Cell culture

The NSCLC cell lines A549, H1650 and the immortalized lung cell line BEAS‐2B were purchased from ATCC (Manassas, VA, USA). Cells were cultured in RPMI‐1640 medium (Gibco, Carlsbad, CA, USA), which was supplemented with 10% fetal bovine serum (FBS, Sigma‐Aldrich, St. Louis, MO, USA) and 1% penicillin–streptomycin in a humidified chamber containing 5% CO_2_ at 37°C. The culture medium was replaced every third day, and cells were sustained in log phase growth.

G‐Rh2 (97% purity, Sigma‐Aldrich) was dissolved in ethanol at the concentration 40 mg/mL. It was stored at −20°C until usage. In brief, cells were grown in 96‐well plates, incubated with ethanol or Rh2 at various concentrations (20, 40, 60 μg/mL) for 24 h.^21^ Subsequently, cells were harvested for following experimentations.

### 2.2 Transient transfection

miR‐negative control (NC) mimic/inhibitor and miR‐28‐5p mimic/inhibitor (GenePharma, Shanghai, China) were transfected into cells by Lipofectamine® 2000 (Thermo Fisher Scientific, Wilmington, DE, USA) for 48 h. subsequently, cells were collected for the following experimentations.

### 2.3 Bioinformatic analysis

The differences of miR‐28‐5p expression profile between lung adenocarcinoma (LUAD) tissues and normal tissues or lung squamous cell carcinoma (LUSC) tissues and normal tissues were analyzed by ENCORI software (http://starbase.sysu.edu.cn/index.php) based on TCGA‐LUAD and TCGA‐LUSC datasets. We have checked the expression data from TCGA database to evaluate the abundances of the miRNAs in current study including miR‐100, miR‐125b, miR‐21, miR‐221, miR‐28‐5p, miR‐31, miR‐365, miR‐424, and miR‐550a. TargetScan V7.2 (http://www.targetscan.org/vert_72/), miRanda (http://www.microrna.org/) and PITA (http://genie.weizmann.ac.il/pubs/mir07/mir07_data.html) software were adopted for the analysis of mRNA targets for miR‐28‐5p, thereafter, the overlapped mRNA targets were subjected to GO analysis by DAVID software (https://david.ncifcrf.gov/summary.jsp). The correlation between STK4 and miR‐28‐5p was studied by ENCORI software based on TCGA‐LUSC dataset. The correlation between miR‐28‐5p/STK4 and OS in LUAD was analyzed by Kaplan–Meier Plotter. immunohistochemistry (IHC) of STK4 expression was analyzed from the HPA database.

### 2.4 Cell counting kit‐8 assay

Cell Counting Kit‐8 (CCK‐8; Dojindo Molecular Technologies, Inc.) was utilized for the examination of cell proliferation. Briefly, cells at the logarithmic phase were seeded in 96‐well plates and grown for 24 h, then subjected to the aforementioned treatments (transient transfection and/or G‐Rh2 treatment). Thereafter, the CCK‐8 solution (10 μL) was added in each well. Cells were incubated for further 2 h prior to the measurement of OD value (450 nm) with an iMark microplate reader (Bio‐Rad, Hercules, CA, USA).

### 2.5 Flow cytometry

Cell apoptotic rates were measured with Annexin V‐fluorescein isothiocyanate (FITC) and propidium iodide (PI) from a Annexin V‐FITC Apoptosis Detection kit (Thermo Fisher Scientific, Inc.). Briefly, cells were subjected to the aforementioned treatments (transient transfection and/or G‐Rh2 treatment), prior to the resuspension in 1× Binding Buffer (100 μL). Subsequently, the cells were added with FITC (5 μL) and PI (5 μL) successively and incubated for 15 min at room temperature. Thereafter, cells were resuspended in 1× Binding Buffer (400 μL), before subjection to flow cytometric analysis (within 1 h after resuspension) by FlowJo 10.7 (FlowJo LLC).

As for cell cycle, cells were harvested and treated with 70% ethanol for 12 h at 37°C. On the next day, cells were stained with PI, then subjected to flow cytometry analysis. Data were analyzed with FlowJo 10.7.

### 2.6 DNA staining by 4, 6‐diamidino‐2‐phenylindole (DAPI)

Cells were fixed by 4% paraformaldehyde (Aladdin, Shanghai, China) for 10 min at room temperature, then rinsed by PBS thrice, and permeabilized by 0.3% Triton X‐100 (Aladdin) for 10 min. Thereafter, cells were rinsed by PBS thrice, next stained with DAPI (Meilune Biotech) for 15 min. At last, cells were captured by a confocal microscope (Zeiss).

### 2.7 qRT‐PCR

Total RNA from tissue samples and cell lines were extracted by miRNeasy Mini Kit (Qiagen). As for the synthesis of cDNA, RNA was reverse transcribed by a SuperScript III First‐Strand kit (Thermo Fisher Scientific, Inc.). Real‐time RT‐PCR assays were performed by SYBR Green PCR Master Mix (Applied Biosystems; Foster, CA, USA) on a 7900HT Fast Real‐Time PCR system (Applied Biosystems). Relative expression of miRNA and mRNA was normalized to U6 and GAPDH, respectively, and analyzed by the 2^−ΔΔCq^ method. The primer sequences are listed in Table 1.

**TABLE 1:** Primer sequences.

Genes	Forward (5′–3′)	Reverse (5′–3′)
GAPDH	GAAGGTGAAGGTCGGAGTC	GAAGATGGTGATGGGATTTC
U6	CTCGCTTCGGCAGCACA	AACGCTTCACGAATTTGCGT
STK4	GCCTCTAAAGATTGCAGCTCCTT	AAGGAGCTGCAATCTTTAGAGGC
miR‐28‐5p	AAGGAGCTCACAGTCTATTGAG	CTCAATAGACTGTGAGCTCCTT
miR‐100	AACCCGTAGATCCGAACTTGTG	CACAAGTTCGGATCTACGGGTT
miR‐125b	TCCCTGAGACCCTAACTTGTGA	TCACTTGTTAGGGTCTCAGGGA
miR‐21	TAGCTTATCAGACTGATGTTGA	TCAACATCAGTCTGATAAGCTA
miR‐221	AGCTACATTGTCTGCTGGGTTTC	GAAACCCAGCAGACAATGTAGCT
miR‐31	AGGCAAGATGCTGGCATAGCT	AGCTATGCCAGCATCTTGCCT
miR‐365	TAATGCCCCTAAAAATCCTTAT	ATAAGGATTTTTAGGGGCATTA
miR‐424	CAGCAGCAATTCATGTTTTGAA	TTCAAAACATGAATTGCTGCTG
miR‐550a	TGTCTTACTCCCTCAGGCACAT	ATGTGCCTGAGGGAGTAAGACA

### 2.8 Western blot

Total proteins from cell lines and tissue samples were extracted by ice‐cold Mammalian Protein Extraction reagent (Thermo Fisher Scientific, Inc.) containing 1% protease inhibitor cocktail (Merck KGaA, Darmstadt, Germany). Protein concentration was assessed by the bicinchoninic acid (BCA) kit (Thermo Fisher Scientific, Inc.). Samples (20 μg protein/lane) were separated by electrophoresis on SDS‐PAGE gel (8 to 10%), followed by transferring onto the polyvinylidene difluoride (PVDF) membrane (Bio‐Rad Laboratories, Inc., Hercules, CA, USA). Thereafter, the PVDF membrane was subjected to blockage by Blocking One (Nacalai Tesque, Kyoto, Japan), incubated with primary antibodies against GAPDH (5174, 1:1000 dilution, Cell Signaling Technology), STK4 (14,946, 1:1000 dilution, Cell Signaling Technology), p‐β‐catenin (9566, 1:1000 dilution, Cell Signaling Technology), and total β‐catenin (8480, 1:1000 dilution, Cell Signaling Technology) at 4°C overnight and horseradish peroxidase (HRP)‐conjugated secondary antibodies (7074, 1:1000 dilution, Cell Signaling Technology) at room temperature for 1 h, successively. Protein bands were visualized by enhanced chemiluminescence (ECL, PerkinElmer, Inc.). Gray density was quantified by Quantity One software (version 4.62; Bio‐Rad Laboratories, Inc.). Relative protein expression was normalized to GAPDH.

### 2.9 Dual luciferase reporter assay

STK4 3′UTR was cloned and reconstructed into pmir‐GLO (Promega, Fitchburg, WI, USA), to obtain pmir‐GLO‐STK4 3′UTR‐wild type (WT). pmir‐GLO‐STK4 3′UTR‐mutant (MUT) was obtained by introduction of two‐site mutations into pmir‐GLO‐STK4 3′UTR‐WT by QuikChange II Site‐Directed Mutagenesis kit (Agilent Technologies, Inc.). Thereafter, pmir‐GLO‐STK4 3′UTR‐WT or pmir‐GLO‐STK4 3′UTR‐Mut and miR‐NC mimic or miR‐28‐5p mimic were co‐transfected into cells, and cultured for 48 h. Then Dual Luciferase Reporter Assay System (Promega) was used for the evaluation of relative luciferase activity. The relative luciferase activity was normalized to *Renilla* luciferase (Promega).

### 2.10 TOP/FOP luciferase reporter assay

pRLTK plasmid and either TOP flash plasmid or FOP flash plasmid were transfected into cells. After cells were cultured for 48 h, Dual Luciferase Reporter Assay System (Promega) was used for the evaluation of relative luciferase activity. Results are expressed as the ratio of TOP Flash activity and FOP Flash activity.

### 2.11 Anticancer effect of G‐Rh2 in nude mice

1 nM miR‐28‐5p antagomir (Ribo Bio, Guangzhou, China) were injected into the tumors to downregulate miR‐28‐5p, while 1 nM micrOFF antagomir NC (Ribo Bio, Guangzhou, China) acted as the control. Animal experiments were approved by the Ethical Committee of Affiliated Hospital of Nanjing University of Chinese Medicine (2022DW‐62‐02). Adult female BALB/c nude mice (6‐week) were fed at Affiliated Hospital of Nanjing University of Chinese Medicine, under the control specific pathogen‐free (SPF) conditions: at temperature of 27 ± 1°C, 12‐h light/dark cycle and *ad libitum* access to autoclaved water and food. A549 cells (250 μL, 3 × 10^6^) which were suspended in the mixture (250 μL) of PBS and Matrigel (BD Biosciences), were subcutaneously inoculated into nude mice. When the tumor size reached 20 mm^3^ (measured by a caliper), mice were treated with micrOFF antagomir NC + vehicle, micrOFF antagomir NC + G‐Rh2^19^ (40 mg/kg, intraperitoneally) or miR‐28‐5p antagomir+G‐Rh2 (40 mg/kg, intraperitoneally) (*n* = 6) every 3 days. Tumor volume (mm^3^) was measured with a caliper and calculated by the formula: 1/2 × length × width.^2^ After tumor growth for 8 weeks, mice were subjected to euthanasia prior to the removement of the tumors. Tumor weight was weighed with an electronic balance.

### 2.12 Statistical analysis

Each experimentation was conducted in triplicate. GraphPad Prism 6 (GraphPad Software, Inc.) was utilized for data analysis. Results were expressed as mean ± SD. Differences between 2 groups were analyzed by unpaired Student's t‐test. Differences among multiple groups were analyzed by one‐way ANOVA followed by Turkey's test, respectively. Pearson correlation analysis was used for analyzing the relationship between miR‐28‐5p and STK4. *p* < 0.05 was deemed as statistically significant.

## 3 RESULTS

### 3.1 G‐Rh2 decreased miR‐28‐5p expression in NSCLC cells

**FIGURE 1:**
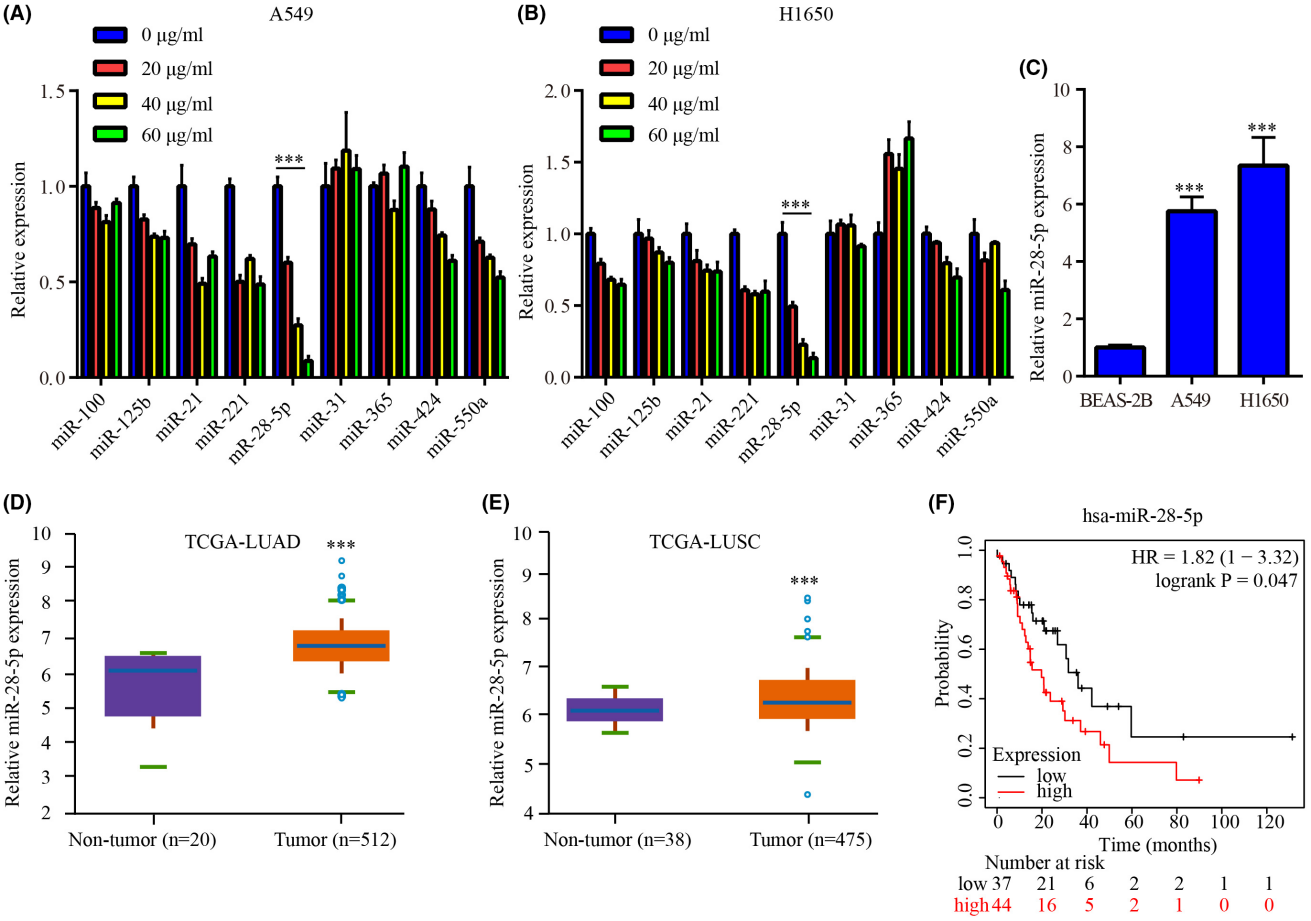
G‐Rh2 reduced the expression of miR‐28‐5p in NSCLC cells. The effects of G‐Rh2 on miR‐28‐5p expression in A549 (A) and H1650 (B) were assessed by RT‐qPCR. The expression profile of miR‐28‐5p in BEAS‐2B and a panel of NSCLC cell lines was assessed by RT‐qPCR (C). The expression profile of miR‐28‐5p in normal tissues and LUAD tissues was analyzed by TCGA‐LUAD dataset (D). The expression profile of miR‐28‐5p in normal tissues and LUSC tissues was analyzed by TCGA‐LUSC dataset (E). The survival plot for miR‐28‐5p in LUAD was analyzed by Kaplan–Meier Plotter (F). **p < 0.01, ***p < 0.001, vs. control, non‐tumor tissues, or BEAS‐2B.

Based on a microarray assay, G‐Rh2 was reported to decrease the expression of 24 miRNAs in A549,^21^ however, some of them were functionally unknown. To further affirm the miRNAs which were regulated by G‐Rh2, the expressions were detected. In A549, miR‐28‐5p, miR‐424, and miR‐550a were observed to be reduced by G‐Rh2 dose‐dependently (20, 40, 60 μg/mL), among which, miR‐28‐5p exhibited the most significant downregulation (Figure 1A). Consistently, in H1650, miR‐28‐5p was downregulated by G‐Rh2 in dose‐dependent manner (20, 40, 60 μg/mL) (Figure 1B). Moreover, we have checked the expression data from TCGA database to evaluate the abundance of these miRNAs, as showed in Table 2, miR‐28 expression was lower compared with highly abundant miRNAs such as miR‐21 but higher than lowly expressed miRNAs such as miR‐550a.

**TABLE 2:** Expression levels of miRNAs in lung cancer from TCGA database.

miRNA	LUAD (log2(RPM))	LUSC (log2(RPM))
miR‐100	13.12452039	12.23364963
miR‐125b	9.15992178	9.19357464
miR‐21	15.12825819	18.29398118
miR‐221	8.310067408	8.393647867
miR‐28‐5p	6.867155163	7.016028242
miR‐31	4.088311236	5.482202926
miR‐365	5.455820365	5.780310099
miR‐424	6.703626771	6.602735976
miR‐550a	1.613531653	1.687060688

By literature reviewing, it was found that the function of miR‐28‐5p in NSCLC has not been uncovered. Firstly, miR‐28‐5p was observed to be increased in NSCLC cell lines (A549, H1650) compared with BEAS‐2B (Figure 1C). By analyzing TCGA‐LUAD dataset, miR‐28‐5p was found to be elevated in 512 LUAD tissues compared with 20 normal tissues (Figure 1D); meanwhile, miR‐28‐5p was increased in 475 LUSC tissues compared with 38 normal tissues according to TCGA‐LUSC dataset (Figure 1E), which was in line with the only one report showing the upregulation of miR‐28 in 478 LUSC tissues compared with 45 normal tissues according to TCGA‐LUSC dataset in 2018.^22^ In addition, miR‐28‐5p was positively associated with poor prognosis of patients in stage III LUAD (Figure 1F). The above findings suggested an oncogenic role of miR‐28‐5p in NSCLC.

### 3.2 Downregulation of miR‐28‐5p induced cell proliferation reduction, cell cycle arrest, and cell apoptosis in NSCLC cells

G‐Rh2 was reported to exhibit inhibitory effect on NSCLC cell growth by inducing cell cycle arrest and cell apoptosis.^18^ In current study, G‐Rh2 decreased miR‐28‐5p expression, which was increased in NSCLC tissues and cell lines; therefore, the corresponding biological functions of miR‐28‐5p inhibitor in NSCLC were explored, to identify whether its functions were consistent with G‐Rh2.

**FIGURE 2:**
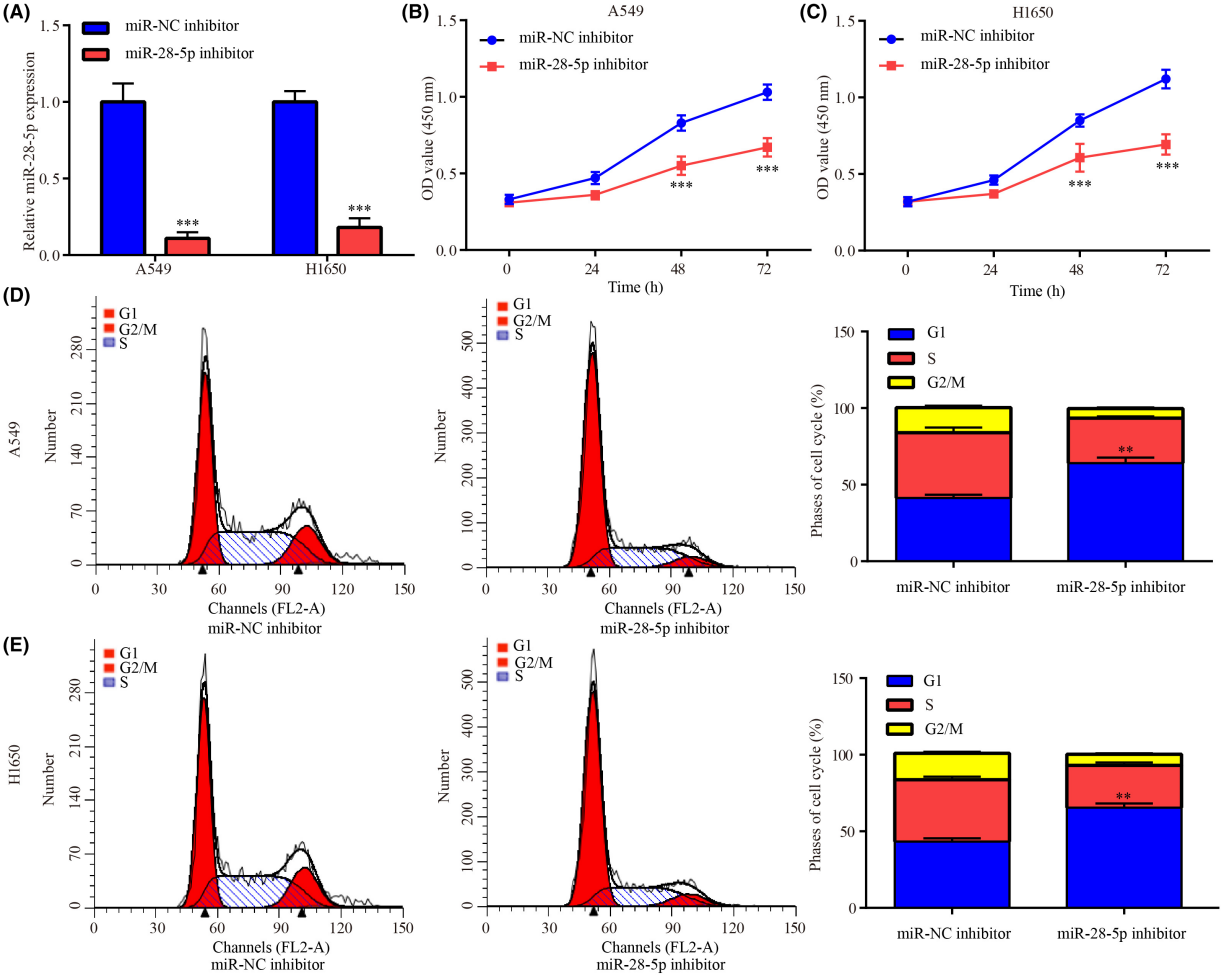
miR‐28‐5p inhibitor inhibited cell proliferation and cell cycle progression in NSCLC. In A549 and H1650, miR‐28‐5p expression in miR‐NC inhibitor group and miR‐28‐5p inhibitor group was assessed by RT‐qPCR (A). The effects of miR‐28‐5p inhibitor in A549 and H1650 cell proliferation (B and C) and cell cycle (D and E) were detected by CCK‐8 and flow cytometry assay, respectively. **p < 0.01, ***p < 0.001, vs. miR‐NC inhibitor.

**FIGURE 3:**
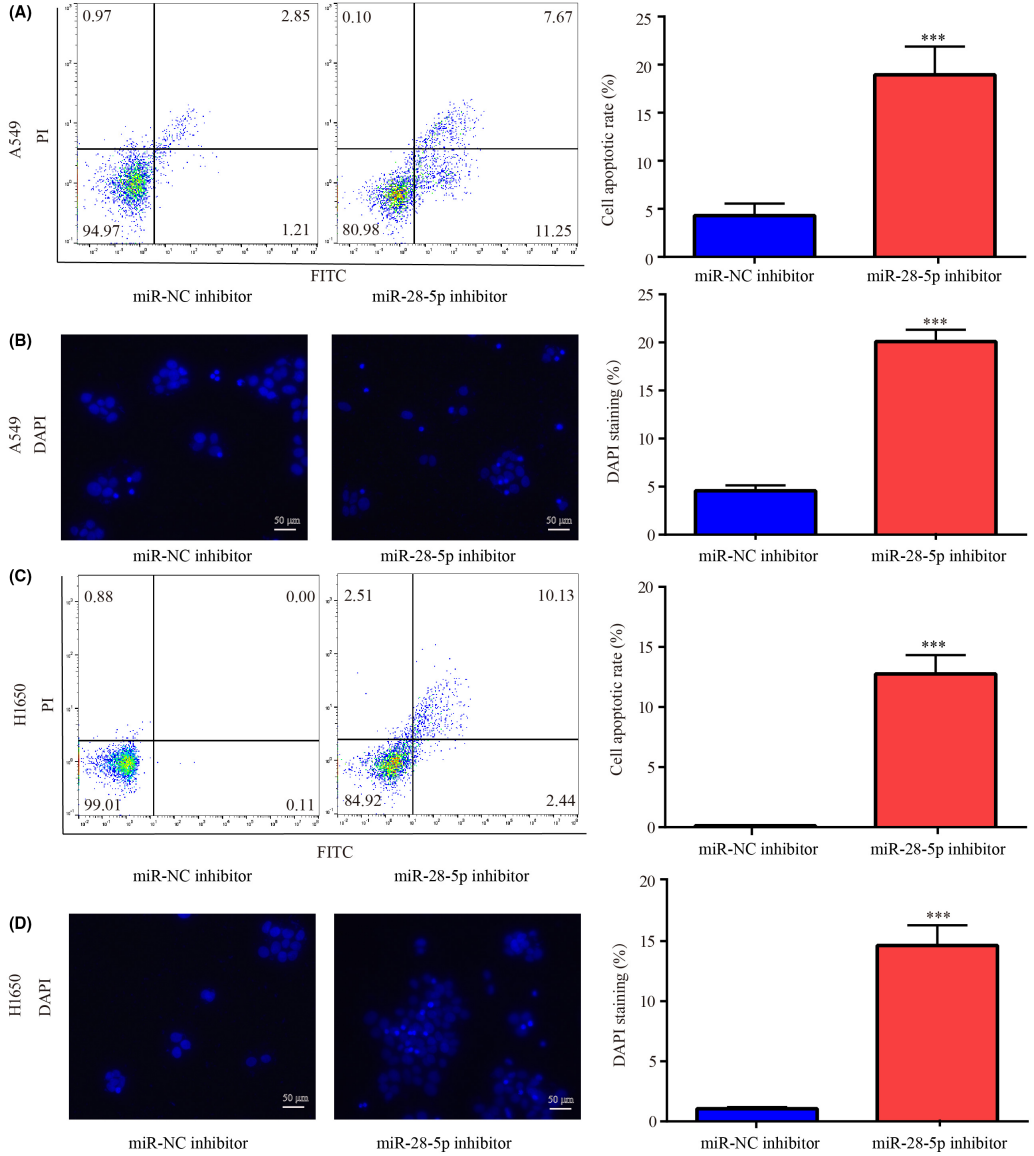
miR‐28‐5p inhibitor‐induced cell apoptosis in NSCLC. The effects of miR‐28‐5p inhibitor in A549 cell apoptosis were detected by flow cytometry assay (A) and DAPI staining (B). The effects of miR‐28‐5p inhibitor in H1650 cell apoptosis were detected by flow cytometry assay (C) and DAPI staining (D). ***p < 0.001, vs. miR‐NC inhibitor.

Following miR‐28‐5p downregulation by miR‐28‐5p inhibitor in A549 and H1650 (Figure 2A), cell proliferation was greatly inhibited (Figure 2B,C), G0/G1 phase arrest (Figure 2D,E) and cell apoptosis (Figure 3A–D) were significantly induced in comparison with miR‐NC inhibitor.

The findings demonstrated that the biological functions of miR‐28‐5p inhibitor were consistent with G‐Rh2, indicating that the downregulation of miR‐28‐5p was indispensable for the anticancer activity of G‐Rh2 in NSCLC.

### 3.3 Downregulation of miR‐28‐5p repressed the activity of Wnt signaling in NSCLC cells

**FIGURE 4:**
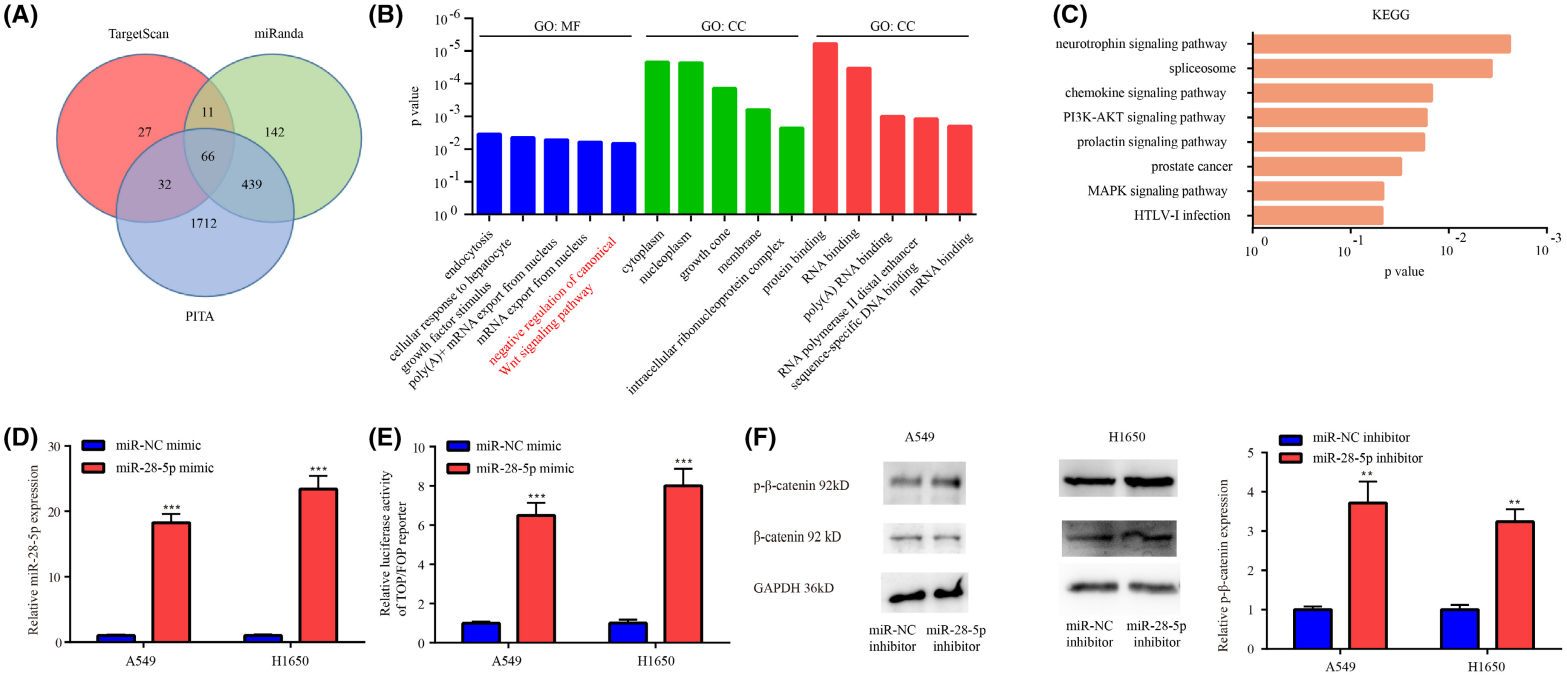
miR‐28‐5p inhibitor inactivated Wnt signaling in NSCLC cells. In total, 140, 658 and 2249 target genes were predicted by TargetScan7.2, miRanda, and PITA software, respectively (A). After GO analysis on the overlapped 66 genes, the top 5 hits of GO_CC, GO_MF, and GO_BP were presented (B). Genes were enriched in oncogenic pathways evidenced by KEGG analysis (C). In A549 and H1650, miR‐28‐5p expression in miR‐NC mimic group and miR‐28‐5p mimic group was assessed by RT‐qPCR (D). TOP/FOP luciferase reporter assay was performed to explore the relation between miR‐28‐5p and Wnt signaling pathway in A549 and H1650 (E). The effect of miR‐28‐5p inhibitor on p‐β‐catenin of A549 and H1650 was examined by western blot (F). **p < 0.01, ***p < 0.001, vs. miR‐NC inhibitor.

miRNAs function by binding to the 3′UTR of target mRNAs.^8^ In total, 140, 658 and 2249 potential target mRNAs were predicted by TargetScan V7.2, miRanda and PITA, respectively; among which, 66 mRNAs were overlapped (Figure 4A). As presented in Figure 4B, GO analysis on the 66 genes showed that, the top 5 hits of GO: (biological process) BP, GO: (cell component) CC, and GO: (molecular function) MF were enriched in negative regulation of canonical Wnt signaling, Cytoplasm and Protein binding. KEGG analysis on the 66 genes exerted that, they were enriched in oncogenic pathways, such as, PI3K‐AKT and MAPK (Figure 4C). Since the activity of Wnt signaling can be inactivated by G‐Rh2 in hepatocellular carcinoma (HCC),^17^ the effects of miR‐28‐5p inhibitor on Wnt signaling pathway were explored in current study to identify whether they were consistent with G‐Rh2.

Following miR‐28‐5p upregulation by miR‐28‐5p mimic in A549 and H1650 (Figure 4D), the luciferase activity in TOP/FOP luciferase reporter assay was increased (Figure 4E). Additionally, p‐β‐catenin level was increased by miR‐28‐5p inhibitor in A549 and H1650 (Figure 4F).

These results collectively indicated that miR‐28‐5p inhibition contributed to the inactivation of Wnt signaling in NSCLC, which was in line with the role of G‐Rh2 in HCC.^17^

### 3.4 miR‐28‐5p activated Wnt signaling by targeting STK4 in NSCLC cells

**FIGURE 5:**
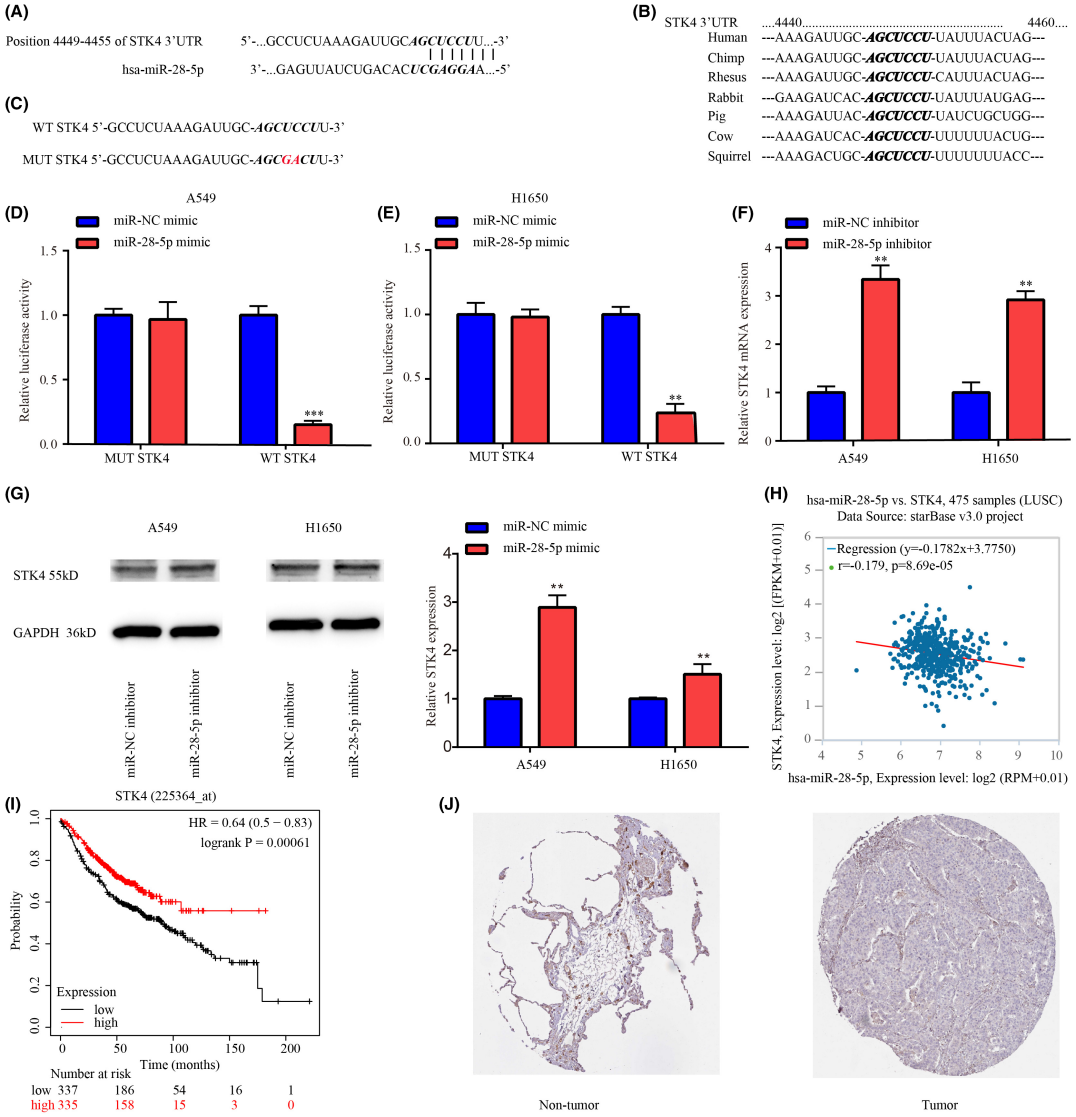
miR‐28‐5p activated Wnt signaling by targeting STK4 in NSCLC cells. The potential binding site between miR‐28‐5p and 3′UTR of STK4 was shown (A). The potential binding site was conserved among Human, Chimp, Rhesus, Squirrel, Rabbit, Pig, Cow, and Cat (B). The WT and Mut forms of STK4 3′UTR were presented (C). Dual luciferase reporter assay was performed to explore the relation between miR‐28‐5p and STK4 in A549 and H1650 (D and E). The effects of miR‐28‐5p inhibitor on STK4 mRNA and protein expression in A549 and H1650 were tested by RT‐qPCR and Western blot (F and G). TCGA‐LUSC cohort was applied to identify the relation between miR‐28‐5p and STK4 in LUSC samples (H). Kaplan–Meier Plotter was conducted to analyze the survival rate for different STK4 expression (high and low) with different prognostic risks (I). IHC of STK4 expression in non‐tumor and tumor tissues was analyzed from the HPA database (J). **p < 0.01, ***p < 0.001, vs. miR‐NC inhibitor.

Among the 66 potential mRNA targets of miR‐28‐5p, STK4 was known as a negative regulator of Wnt signaling pathway and a tumor suppressor in NSCLC.^23^ By sequence alignment, a potential binding site between miR‐28‐5p and 3′UTR of STK4 mRNA was observed (Figure 5A), which was conserved among numerous species including Human, Chimp, Rhesus, Squirrel, Rabbit, Pig, Cow, and Cat (Figure 5B). WT and the MUT forms of STK4 3′UTR were presented (Figure 5C). The relative luciferase activity of WT STK4 3′UTR but not MUT STK4 3′UTR was repressed by miR‐28‐5p mimic in A549 and H1650 (Figure 5D,E). Furthermore, STK4 mRNA and protein expressions were dramatically increased by miR‐28‐5p inhibitor in A549 and H1650 (Figure 5F,G). More importantly, in 475 LUSC samples from TCGA‐LUSC cohort, STK4 mRNA was negatively correlated with miR‐28‐5p level (Figure 5H). Moreover, in LUAD, higher STK4 expression was positively related with the prolong OS (Figure 5I), weak STK4 stain was observed in normal tissues, while there was non‐STK4 stain in tumor tissues (Figure 5J).

Thus, the data indicated for the first time that SKT4 was a target of miR‐28‐5p in NSCLC.

### 3.5 G‐Rh2 inactivated Wnt signaling by downregulation of miR‐28‐5p in NSCLC cells

**FIGURE 6:**
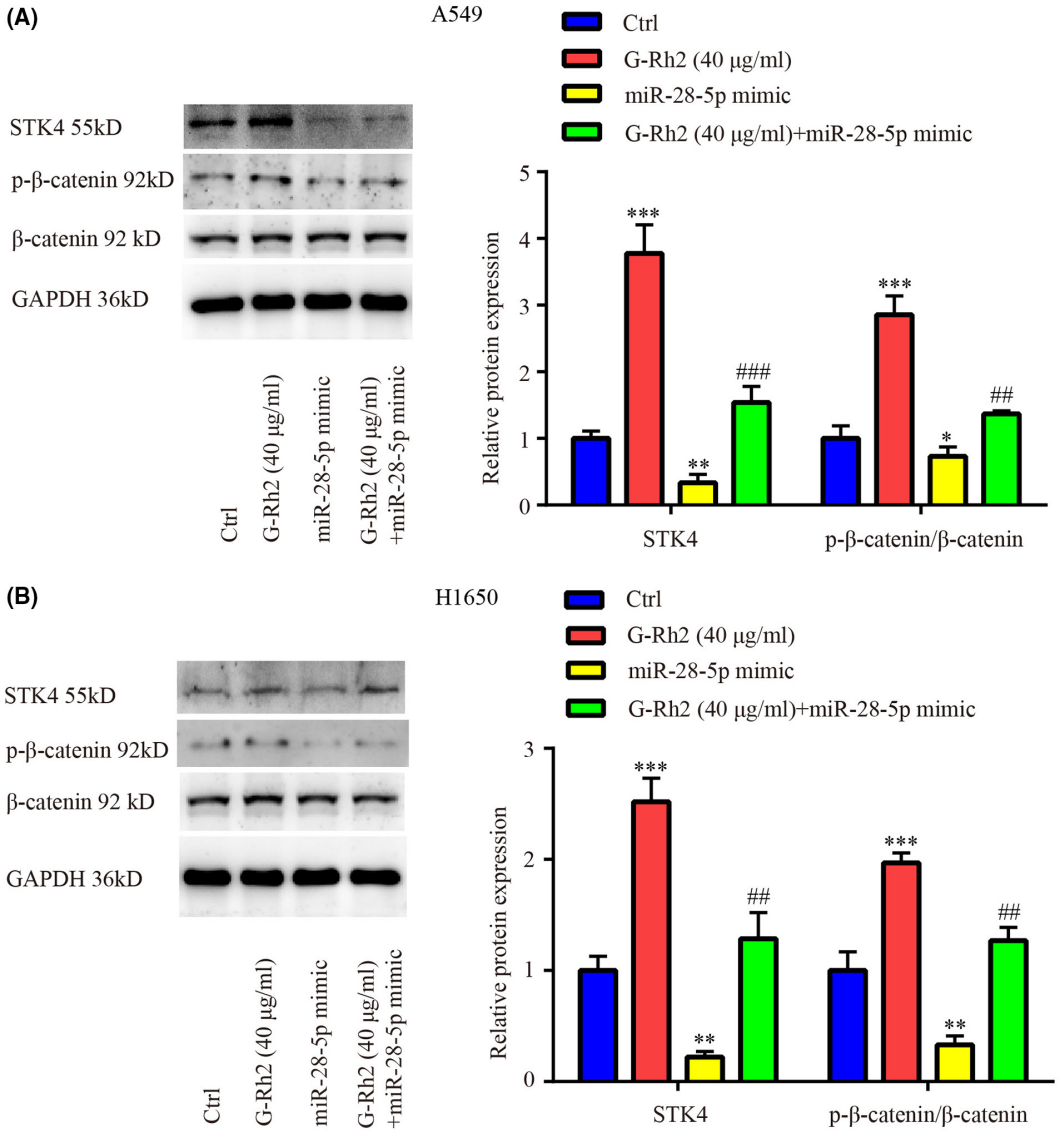
G‐Rh2 inactivated Wnt signaling by downregulating miR‐28‐5p in NSCLC. The effects of G‐Rh2, miR‐28‐5p mimic and G‐Rh2 + miR‐28‐5p mimic on STK4 and p‐β‐catenin protein levels in A549 and H1650 were evaluated by western blot (A and B). *p < 0.05, **p < 0.01, ***p < 0.001, vs. control. ^##^ *p* < 0.01, ^###^ *p* < 0.001, vs. G‐Rh2.

A549 and H1650 were treated with G‐Rh2^21^ or miR‐28‐5p mimic or G‐Rh2 + miR‐28‐5p mimic. In A549 and H1650, G‐Rh2 increased STK4 and p‐β‐catenin level, miR‐28‐5p mimic alone showed inhibitory effect on STK4 and little inhibitory effect on p‐β‐catenin level, however, miR‐28‐5p mimic antagonized the effect of G‐Rh2 on STK4 and p‐β‐catenin level; meanwhile, there was no significant difference in β‐catenin level among the 4 groups (Figure 6A,B).

Altogether, we suggested the antagonistic role between G‐Rh2 and miR‐28‐5p mimic in Wnt signaling pathway of NSCLC.

### 3.6 G‐Rh2 induced cell proliferation reduction, cell cycle arrest and cell apoptosis of NSCLC cells by downregulation of miR‐28‐5p

**FIGURE 7:**
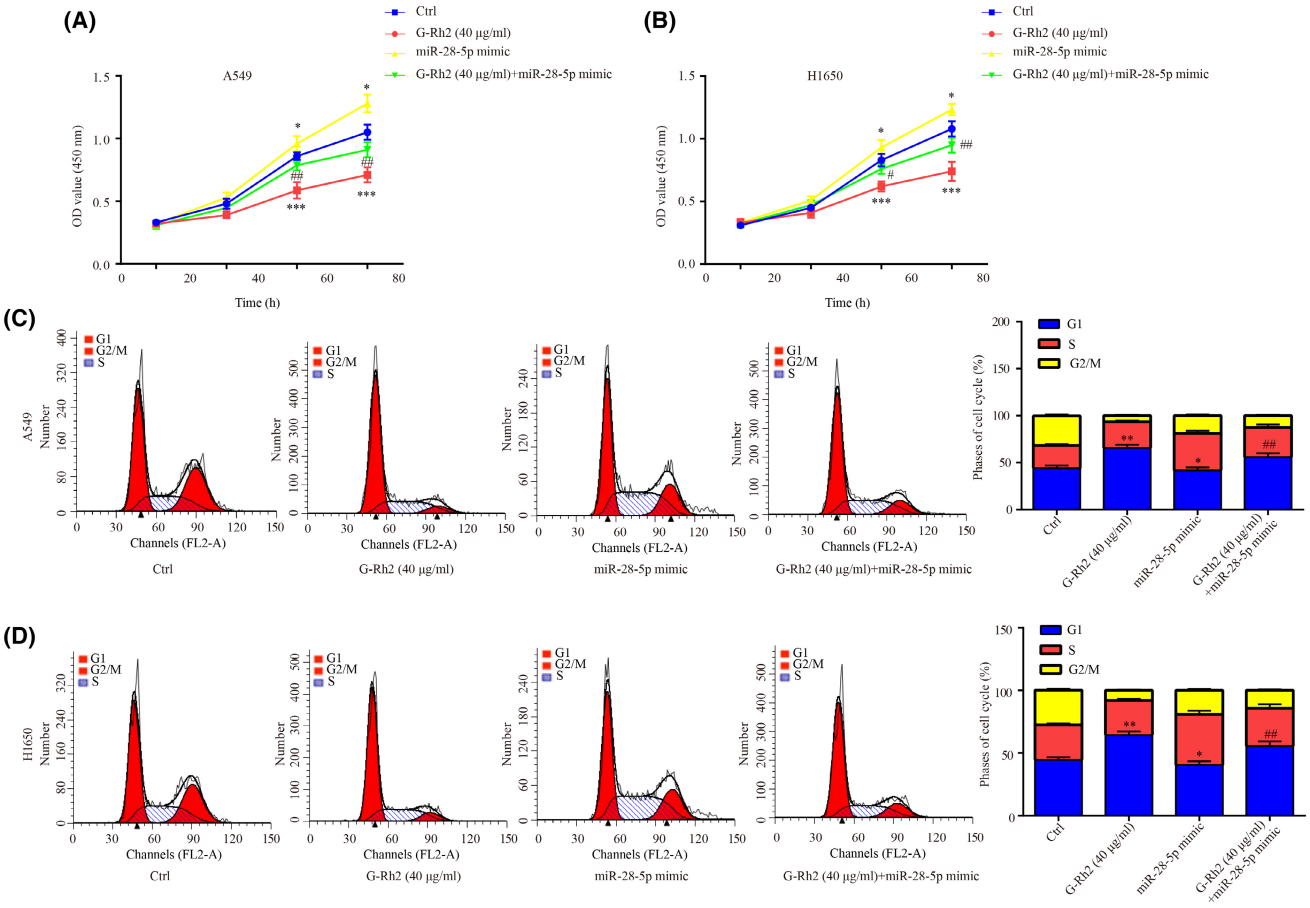
G‐Rh2 inhibited NSCLC cell proliferation and cell cycle progression by downregulating miR‐28‐5p. The effects of G‐Rh2, miR‐28‐5p mimic, and G‐Rh2 + miR‐28‐5p mimic on cell proliferation (A and B) and cell cycle (C and D) in A549 and H1650 were evaluated by CCK‐8 and flow cytometry, respectively. *p < 0.05, **p < 0.01, vs. control. ^#^ *p* < 0.05, ^##^ *p* < 0.01, vs. G‐Rh2.

**FIGURE 8:**
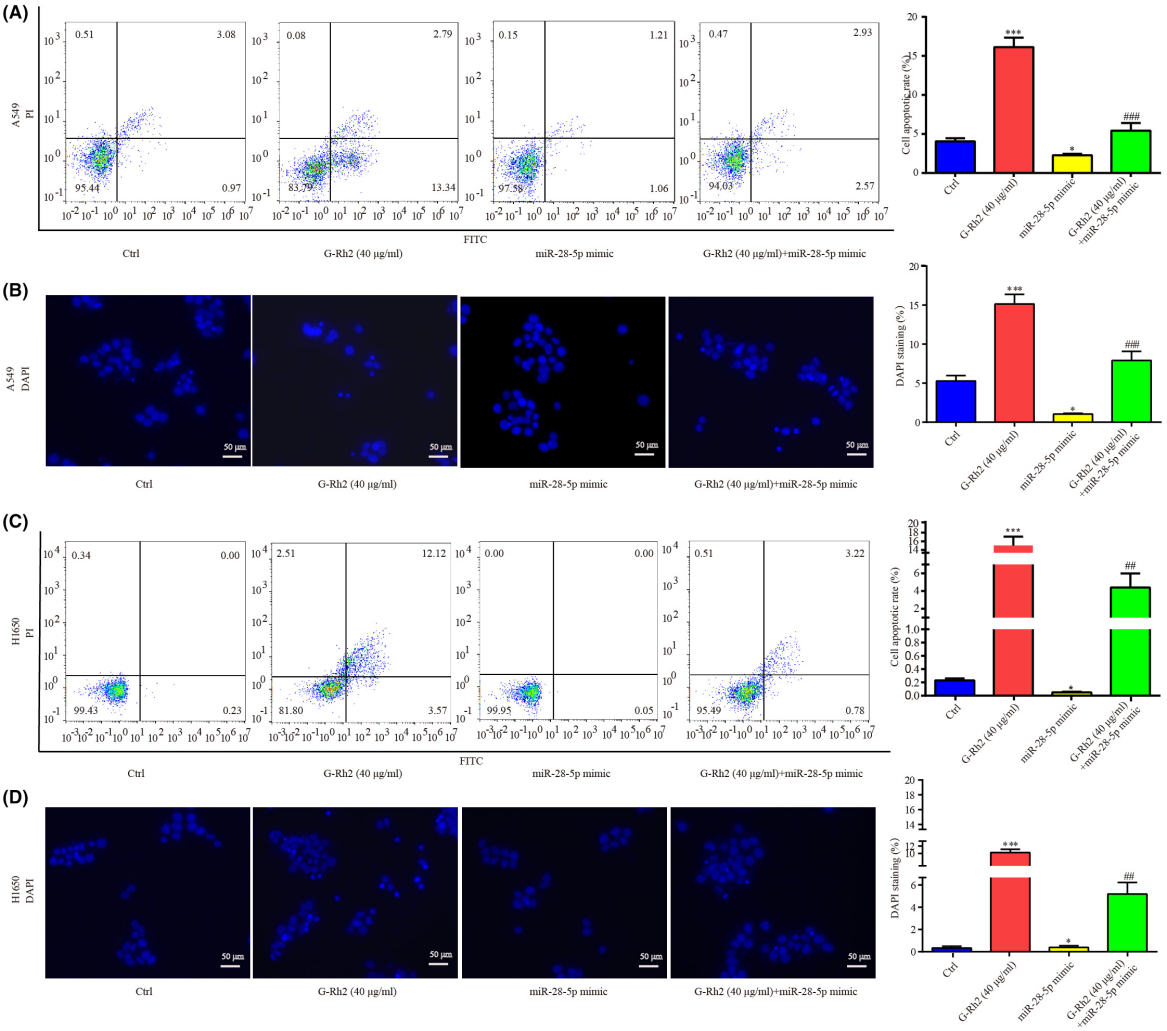
G‐Rh2 induced NSCLC cell apoptosis by downregulating miR‐28‐5p. The effects of G‐Rh2, miR‐28‐5p mimic and G‐Rh2 + miR‐28‐5p mimic on cell apoptosis in A549 were evaluated by flow cytometry (A) and DAPI staining (B). The effects of G‐Rh2, miR‐28‐5p mimic, and G‐Rh2 + miR‐28‐5p mimic on cell apoptosis in H1650 were evaluated by flow cytometry (C) and DAPI staining (D). *p < 0.05, ***p < 0.001, vs. control. ^##^ *p* < 0.01, ^###^ *p* < 0.001, vs. G‐Rh2.

A549 and H1650 were treated with G‐Rh2^21^ or miR‐28‐5p mimic or G‐Rh2 + miR‐28‐5p mimic. It was found that, G‐Rh2 greatly suppressed cell proliferation, miR‐28‐5p mimic showed a weak pro‐proliferation effect, while miR‐28‐5p mimic attenuated the anti‐proliferation effect of G‐Rh2 in A549 and H1650 (Figure 7A,B). Additionally, G‐Rh2 led to cell arrest at G0/G1 phase (Figure 7C,D) and cell apoptosis (Figure 8A–D), which were reversed by co‐administration of miR‐28‐5p mimic in A549 and H1650.

Collectively, we demonstrated the antagonistic role between G‐Rh2 and miR‐28‐5p mimic in NSCLC cell proliferation, cell cycle and cell apoptosis.

### 3.7 G‐Rh2 and miR‐28‐5p antagomir showed synergistic effect on suppression of NSCLC tumor growth and inactivation of Wnt signaling in vivo

**FIGURE 9:**
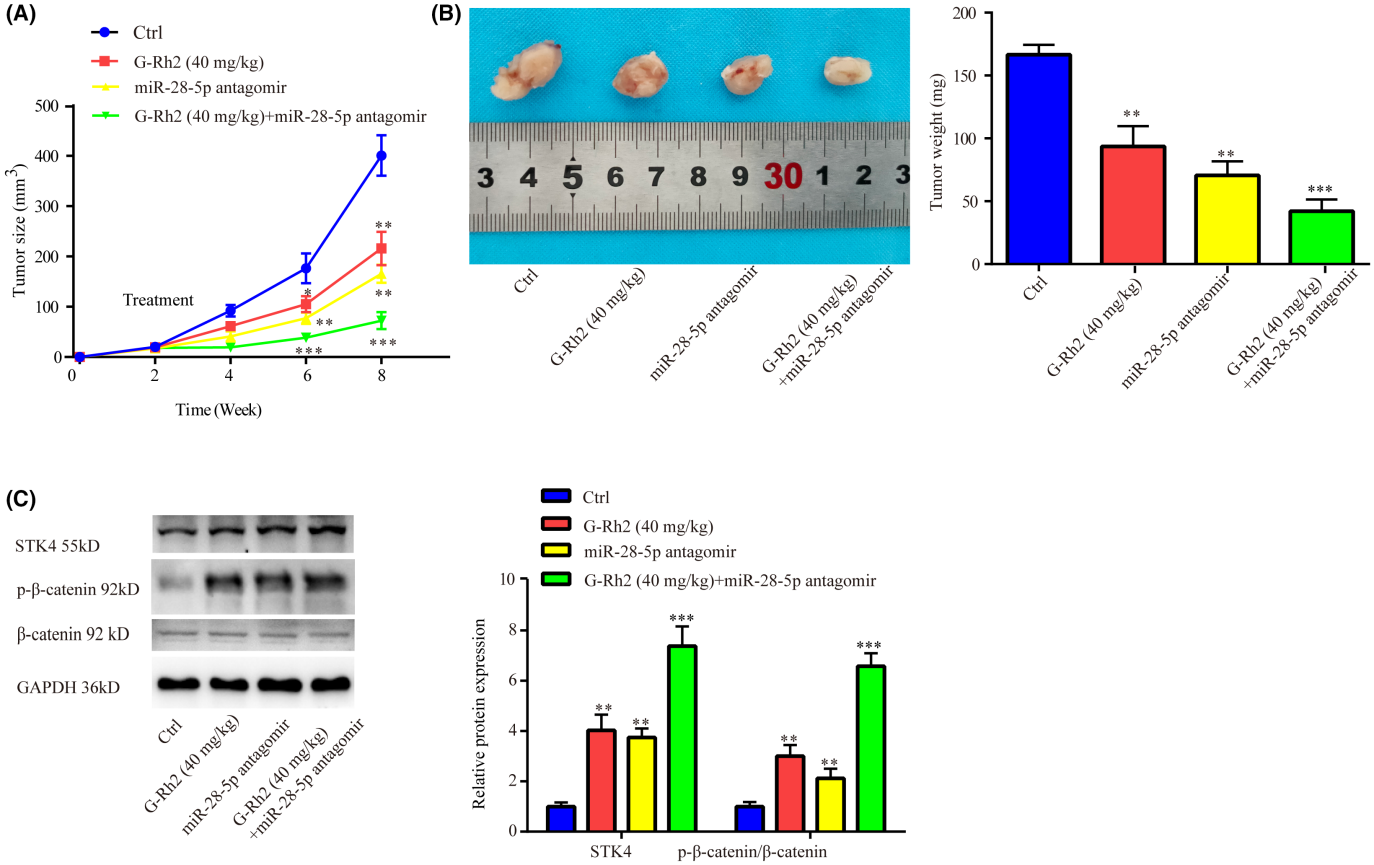
G‐Rh2 and miR‐28‐5p antagomir exhibited synergistic effect on inhibiting NSCLC tumor growth in vivo. The effects of G‐Rh2, miR‐28‐5p antagomir, and G‐Rh2 + miR‐28‐5p antagomir on tumor size and weight were investigated in nude mice (A and B). The effects of G‐Rh2, miR‐28‐5p antagomir and G‐Rh2 + miR‐28‐5p antagomir on SKT4 and p‐β‐catenin protein levels were assessed by western blot (C). *p < 0.05, **p < 0.01, ***p < 0.001, vs. control.

The above results implied that miR‐28‐5p was indispensable for function of G‐Rh2 in NSCLC in vitro. To investigate their relation in vivo, nude mice were injected with A549 cells, then treated with G‐Rh2^19^ or miR‐28‐5p antagomir or G‐Rh2 + miR‐28‐5p antagomir. The size and weight of tumors were reduced by G‐Rh2 or miR‐28‐5p antagomir alone, especially G‐Rh2 + miR‐28‐5p antagomir (Figure 9A,B). Protein expressions of SKT4 and p‐β‐catenin in tumors were increased by G‐Rh2 or miR‐28‐5p antagomir alone, especially G‐Rh2 + miR‐28‐5p antagomir; however, there was no significant difference in β‐catenin level among the 4 groups (Figure 9C).

In conclusion, we proved the synergistic role of G‐Rh2 and miR‐28‐5p antagomir in NSCLC tumor growth and Wnt signaling pathway.

## 4 DISCUSSION

Current therapies which mainly consist of chemotherapy, radiotherapy, and the emerging target therapy, exert unsatisfactory effectiveness in patients with NSCLC.^24^ Several studies have reported the anticancer activity of G‐Rh2 in NSCLC, for example, the combination of betulinic acid, parthenolide, honokiol, and G‐Rh2 exerts a synergistic effect on the treatment of lung cancer,^25^ Aidi injection which consists of Ginseng extracts, in combined with paclitaxel‐based chemotherapy improve the clinical efficacy and quality of life (QOL) in patients at stage III/IV NSCLC.^26^ However, the underlying precise molecular mechanisms of G‐Rh2 in NSCLC remain intricate.

Based on a previous microRNA array, several miRNAs were found to be downregulated by G‐Rh2 in A549.^21^ To affirm the definitive miRNAs which were downregulated by G‐Rh2 in NSCLC, we performed a RT‐qPCR based in‐house screen, indicating that among all the downregulated miRNAs, miR‐28‐5p showed the most downregulation. As for the role of miR‐28‐5p in cancers, it is known that, miR‐28‐5p functions as distinct roles based on different cancer backgrounds, that is, an oncogene or a tumor suppressor. For instance, miR‐28‐5p reduces cell proliferation, while induces cell cycle arrest and apoptosis in nasopharyngeal cancer,^27^ LOXL1‐AS1 promotes the development of pancreatic cancer by sponging miR‐28‐5p/SEMA7A axis,^28^ high miR‐28‐5p expression predicts poor OS in patients with non‐metastatic colorectal cancer.^29^ However, the function of miR‐28‐5p in NSCLC has not been explored yet until now. Herein, an upregulation of miR‐28‐5p was observed in NSCLC cell lines and tumor tissues, whose downregulation reduced cell proliferation, induced cell apoptosis and re‐distribution of cell cycle, suggesting the oncogenic role of miR‐28‐5p in NSCLC for the first time. Consistently, G‐Rh2 also inhibited NSCLC cell proliferation, induced cell cycle arrest and cell apoptosis,^18^ which implied that miR‐28‐5p was responsible for the function of G‐Rh2 in NSCLC.

miRNAs function by binding to the 3′UTR of target mRNAs.^8^ In the present study, using three bioinformatic tools, 66 overlapped mRNAs were predicted as putative targets of miR‐28‐5p. GO analysis exerted that the 66 mRNAs were enriched in the negative regulation of Wnt signaling pathway, which was critical for the development of NSCLC,^22^ and could be inactivated by G‐Rh2 in HCC.^17^ Furthermore, Wnt signaling pathway was firstly verified to be inactivated by miR‐28‐5p inhibitor in NSCLC in current study.

Moreover, among the 66 potential mRNA targets of miR‐28‐5p, STK4 which was a known negative regulator of Wnt signaling pathway and a tumor suppressor in NSCLC,^22^, ^30^ attracted our attentions. Thereafter, STK4 was proved to be targeted by miR‐28‐5p and upregulated by G‐Rh2 in NSCLC cells. As acknowledged, STK4 is a pharmacological target for the improvement of tissue repair and regeneration.^31^ Recently, it has shown the potential to be developed as a promising therapeutic target in NSCLC. For example, STK4 exerts an antiproliferative effect in NSCLC in vitro and in vivo, which attributes to the induction of apoptosis,^32^ STK4 promotes cell apoptosis via enhancing mitochondrial damage by ROCK1/F‐actin signaling pathways in NSCLC,^33^ genetic deletion of Mst1/2 from a novel transgenic mouse model induces aggressive NSCLC in the lungs.^34^ In the present study, according to other researchers' experiences with excellent screening methods for bioinformatics of LUAD,^35^ higher STK4 expression was observed to be positively related to the prolong OS, lower STK4 expression was observed in tumor tissues compared with normal tissues, we also proved the interaction of miR‐28‐5p‐STK4 and G‐Rh2‐STK4 in NSCLC for the first time.

In addition, G‐Rh2 inactivated Wnt signaling, upregulated STK4 expression, inhibited cell viability and cell cycle, and enhanced cell apoptosis, which was attenuated by miR‐28‐5p mimic in NSCLC cells, suggesting that G‐Rh2 exerted its anti‐proliferation effect by affecting miR‐28‐5p/STK4 axis and the following inactivation of Wnt signaling. Interestingly, G‐Rh2 greatly suppressed cell proliferation and miR‐28‐5p mimic only slightly increased cell proliferation; however, miR‐28‐5p mimic could largely reverse the effects of G‐Rh2, which may attribute to the following reason as shown in Figure 1, miR‐28‐5p is highly expressed in NSCLC cells, we speculated that this high expression of miR‐28‐5p facilitated NSCLC cell proliferation. However, the effect of forced miR‐28‐5p overexpression was weakened as the cancer cells might adapted to the miR‐28‐5p level. Moreover, co‐administration of G‐Rh2 and miR‐28‐5p antagomir exhibited higher inhibitory effects on tumor growth, and inductive effects on STK4 and p‐β‐catenin level, indicating the synergistic role of G‐Rh2 and miR‐28‐5p antagomir in NSCLC tumor growth in vivo by upregulating STK4 and inactivating Wnt signaling. The aforementioned findings further exhibited the involvement of miR‐28‐5p/STK4 axis and Wnt signaling in the anti‐tumor effects of G‐Rh2 in NSCLC.

Taken together, we shed lights on that G‐Rh2 attenuates the development of NSCLC by affecting miR‐28‐5p/STK4 axis and inactivation of Wnt signaling pathway. And we project out a novel therapeutic target for NSCLC.

## AUTHOR CONTRIBUTIONS

**Jun Ma:** Conceptualization (equal); data curation (equal); formal analysis (equal); funding acquisition (supporting); investigation (equal); methodology (equal); project administration (equal). **Di Zhao:** Conceptualization (equal); data curation (equal); formal analysis (equal); investigation (equal); methodology (equal); project administration (equal). **Dahai Yu:** Conceptualization (supporting); data curation (supporting); formal analysis (supporting); investigation (supporting); methodology (supporting). **Wei Song:** Conceptualization (supporting); data curation (supporting); formal analysis (supporting); investigation (supporting). **Xiaofang Yang:** Conceptualization (supporting); investigation (supporting); methodology (supporting); project administration (supporting). **Haitao Yin:** Conceptualization (equal); resources (lead); supervision (lead); validation (lead); visualization (lead); writing – original draft (lead); writing – review and editing (lead).

## FUNDING INFORMATION

Financial support for this work was provided by the National Natural Science Foundation of China (81,672,973, 81,703,758), General project of Jiangsu Provincial Health Commission (M2022061), Medical leaders Foundation of Xuzhou City (XWRCHT20210023), Science and technology program projects of Xuzhou (KC21188), and Beijing Great Medical Public Welfare Foundation Project (DY‐Tumor2022‐J003).

## CONFLICT OF INTEREST STATEMENT

The authors declare no conflict of interest and no competing interests.

## Data Availability

They are available for the corresponding author on special request.
